# Effects of Deep Tillage and Straw Returning on Soil Microorganism and Enzyme Activities

**DOI:** 10.1155/2014/451493

**Published:** 2014-03-26

**Authors:** Baoyi Ji, Hao Hu, Yali Zhao, Xinyuan Mu, Kui Liu, Chaohai Li

**Affiliations:** ^1^Agronomy College, Henan Agricultural Universtiy, Zhengzhou 450002, China; ^2^Institute of Horticulture, Henan Academy of Agricultural Sciences, Zhengzhou 450002, China; ^3^Institute of Digital Agriculture, Zhejiang Academy of Agricultural Sciences, Hangzhou, Zhejiang 310021, China; ^4^Department of Engineering, Nova Scotia Agricultural College, Truro, NS, Canada B2N 5E3

## Abstract

Two field experiments were conducted for two years with the aim of studying the effects of deep tillage and straw returning on soil microorganism and enzyme activity in clay and loam soil. Three treatments, (1) conventional tillage (CT), shallow tillage and straw returning; (2) deep tillage (DT), deep tillage and straw returning; and (3) deep tillage with no straw returning (DNT), were carried out in clay and loam soil. The results showed that deep tillage and straw returning increased the abundance of soil microorganism and most enzyme activities. Deep tillage was more effective for increasing enzyme activities in clay, while straw returning was more effective in loam. Soil microorganism abundance and most enzyme activities decreased with the increase of soil depth. Deep tillage mainly affected soil enzyme activities in loam at the soil depth of 20–30 cm and in clay at the depth of 0–40 cm. Straw returning mainly affected soil microorganism and enzyme activities at the depths of 0–30 cm and 0–40 cm, respectively.

## 1. Introduction

In China, traditional tillage in grain production areas was shallow ploughed to the depth of about 0–20 cm. This may result in soil hardpan layer and affect crop growth and production. Farmers also had the tradition of straw returning, but recently most straws were burned for labor saving, which was a waste of resources and caused environment pollution. It has been accepted that deep tillage and straw returning were significant for the improvement of soil characteristics and agricultural sustainable development. Deep tillage could remediate subsoil compaction, break up high-density soil layer [1], improve water infiltration, change the soil aggregate size distribution [2], enhance root growth and development, and increase crop production potential [3]. Crop straw in the soil surface could moderate soil temperature and increase water infiltration and soil organic carbon [4].

Soil microorganism and enzyme activity are important indicators of soil quality [5]. The change of soil physicochemical characters may directly influence soil microorganism and enzyme activity. As is known, soil microorganism and enzyme activity could activate potential soil nutrient and increase crop yield. Bacteria, actinomycetes, and fungi are the main soil microorganisms. They can decompose organic residues, produce antibiotics, and supply food sources for organisms [6]. Soil microorganisms help to reduce crop residues and biochemically process nutrients to improve the soil. They are major sources of soil enzymes which seem to be related to agriculture management practices. Enzymes play an important role in the nutrients cycling. Benítez et al. thought it can be used as an indicator of soil microbial activity and fertility [7]. On the other hand, enzymes were involved in soil mineralization processes and related to some soil biological properties [8]. Soil microorganism and enzyme activity profiles reflect an important part of plants and soil, which is in close relation to agriculture practice.

Previous studies indicated that tillage and straw returning had great effect on soil microbial community and enzyme activity. Govaerts et al. found that residue application increased the soil microbial community [9]. In many cases, bacteria and fungi under no-tillage were more abundant than conventional tillage [10]. Spedding et al. found the residue in no-tillage systems was mainly decomposed by the fungal community [11]. Deng and Tabatabai reported that activities of phosphatases in no-till/double mulch were significantly greater than those of other treatments studied [12].

However, the influence of deep tillage on soil microbial community and enzyme activity was seldom studied. Soil texture had marked influence on the structure and activity of microbial population and mineralization of carbon. The direct effects of deep tillage and straw returning in different soil types on microorganism and enzyme activities were not known. This study investigated the effect of deep tillage and straw returning on soil microorganism (actinomycete, bacteria, and fungi) and enzyme activities (catalase, phosphatase, urease, and saccharase) in clay and loam soil. The objective was to reveal the direct response of soil microbial community and enzyme activity to deep tillage and straw returning and obtain the internal function mechanism for future agricultural guidance.

## 2. Materials and Methods

### 2.1. Study Sites

Two field experiments were established during the maize growing seasons of 2010 and 2011 on the farm of Hebi County Academy of Agricultural Science (35°67′ N, 114°98′ E) and Luohe County Academy of Agricultural Science (33°57′ N, 113°98′ E), Henan province, China. Both sites have a continental monsoon type climate. The soils of Hebi and Luohe were classified as loam and clay soil according to the USA soil taxonomy method (Soil Survey Staff, USA, 1999). The data of monthly average temperature and rainfall during the study period were collected from a weather station adjacent to the experimental fields and were presented in Figure 1 of [13]. Soil samples were collected at the beginning of experiment. The main physical and chemical properties of soils in the two study sites were presented in Table 1 of [13].

### 2.2. Experiment Designs

The experiment was designed as a randomized block with three replications. Three treatments were set up: (1) conventional tillage (CT), moldboard ploughed to a depth of 20 cm, and straw was returned; (2) deep tillage (DT), moldboard ploughed to a depth of 30 cm, and straw was returned; and (3) deep tillage and straw removed (DNT), moldboard ploughed to a depth of 30 cm, and straw was removed. The crop rotation consisted of winter wheat and summer maize. Soil tillage was carried out after the harvest of maize and before the winter wheat sowing. Residues of postharvest winter wheat were left on the soil surface throughout the study periods. Maize residues were ploughed into the soil with tillage in the CT and DT treatments.

Each treatment plot had 60 m × 20 m area. The summer maize hybrid Zhengdan 958 was sown at a density of 67,500 plant ha^−1^ on June 7 at Hebi and June 5 at Luohe during the two years. Basal fertilizers of 135 kg N ha^−1^ as urea, 135 kg P_2_O_5_ ha^−1^ as diammonium phosphate, and 180 kg K_2_O ha^−1^ as potassium sulfate were applied just before sowing according to N, P, and K content in the soil. 135 kg N ha^−1^ as urea was applied at 12-leaf full expansion. The same culture practices were implemented to all the experimental plots.

### 2.3. Measurements of Soil Microbial Abundances and Enzyme Activities

Soil samples were taken from selected plots with three replications from the soil profile depths of 0–10 cm, 10–20 cm, 20–30 cm, and 30–40 cm in the grain physiological maturity stage (in October) over the 2010-2011 time periods. The soil samples were sieved through a 2 mm sieve and stored in a refrigerator at 4°C. Soil microbial functional groups were analyzed using the most probable number method described by previous studies [14].

Soil urease activity was measured using indophenol colorimetry method with urea as the substrate. Briefly, ammonium was released over 1 h and assayed colorimetrically at 578 nm. Soil urease activity was expressed as mg NH_3_–N g^−1^ dry soil. Saccharase activity was determined by 3,5-dinitrosalicylic acid colorimetry method using sucrose as the substrate. It was expressed as mg glucose g^−1^ dry soil. Soil phosphatase activity was determined with disodium phenyl phosphate colorimetry according to Ge et al. [15], and catalase activity was determined according to Johnson and Temple [16]. The enzyme activities were expressed as mg p-nitrophenol released g^−1^ dry soil in the case of phosphatase and as *μ*mol KMnO_4_ g^−1^ dry soil min^−1^ for catalase. All determinations of enzymatic activities were performed in triplicate, with values reported as means.

### 2.4. Statistical Analysis

Analyses were performed in three replicates, and average values were presented. Analysis of variance (ANOVA) was conducted using SPSS 17.0 software (IBM SPSS, Inc., 2009). Differences between the two years, tillage systems, and soil textures were compared by the Student *t*-test. Statistically significant differences among the different soil depths were determined by Duncan's test. Significant differences were accepted at *P* ≤ 0.05 level and indicated in different letters unless otherwise stated. The same letters in the graph and columns of tables represented no significant difference statistically (*P* > 0.5).

## 3. Results and Discussion

### 3.1. Effects of Deep Tillage and Straw Returning on Soil Microorganism

**Figure 1:**
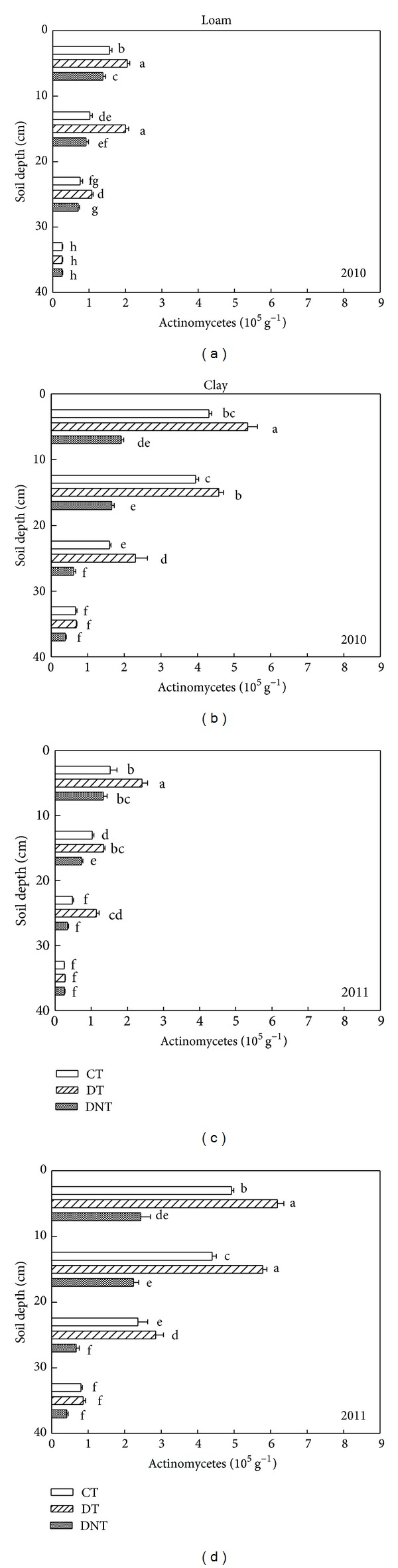
Effects of tillage and straw returning on the number of soil actinomycetes. Different small letters indicated significant difference at *P* < 0.05 according to Duncan's test. CT represented conventional tillage, moldboard ploughed to a depth of 20 cm, and straw was returned. DT represented deep tillage moldboard ploughed to a depth of 30 cm and straw was returned. DNT represented deep tillage moldboard ploughed to a depth of 30 cm and straw was removed.

**Figure 2:**
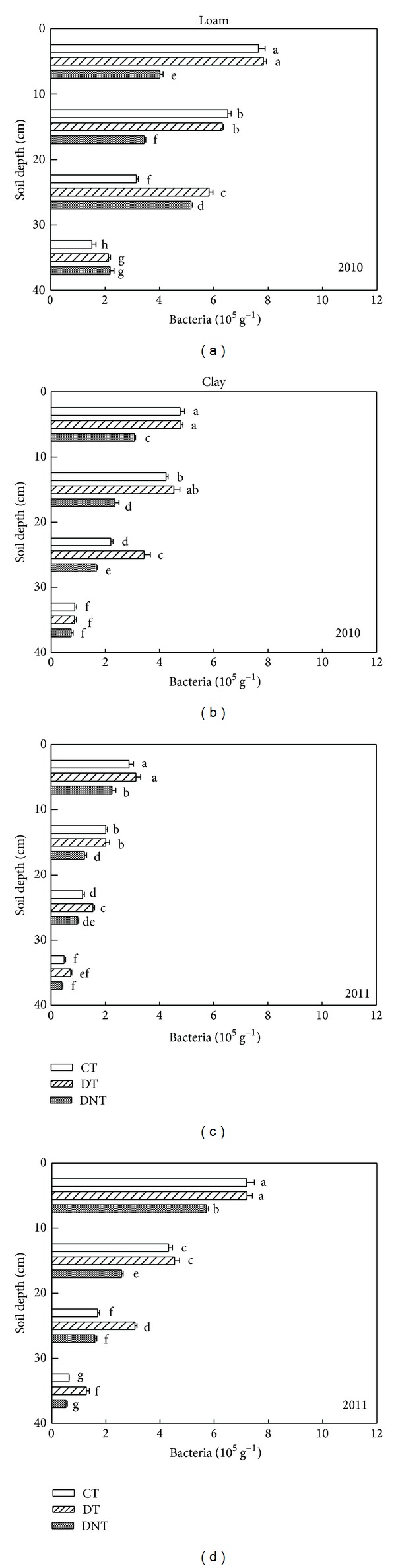
Effects of tillage and straw returning on the number of soil bacteria. Different small letters indicated significant difference at *P* < 0.05 according to Duncan's test. CT represented conventional tillage, moldboard ploughed to a depth of 20 cm, and straw was returned. DT represented deep tillage moldboard ploughed to a depth of 30 cm and straw was returned. DNT represented deep tillage moldboard ploughed to a depth of 30 cm and straw was removed.

**Figure 3:**
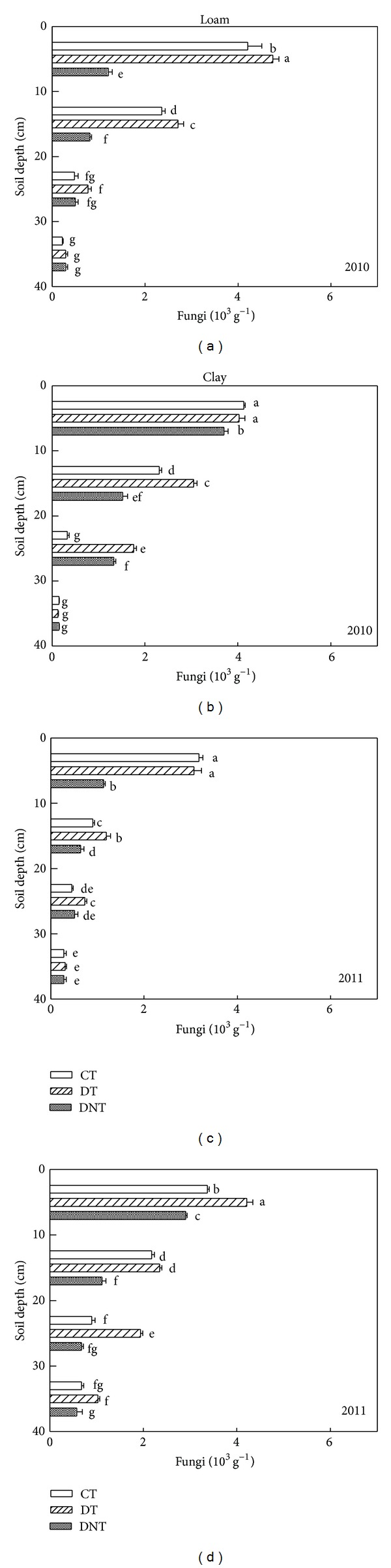
Effects of tillage and straw returning on the number of soil fungi. Different small letters indicated significant difference at *P* < 0.05 according to Duncan's test. CT represented conventional tillage, moldboard ploughed to a depth of 20 cm, and straw was returned. DT represented deep tillage moldboard ploughed to a depth of 30 cm and straw was returned. DNT represented deep tillage moldboard ploughed to a depth of 30 cm and straw was removed.

Tillage and straw returning had great influence on soil microorganism. The abundance of soil microorganism (actinomycetes, bacteria, and fungi) under CT, DT, and DNT conditions in loam and clay was shown in Figures 1, 2, and 3. Seen from the above figures and Table 1, the number of soil actinomycetes, bacteria, and fungi followed the order of DT > CT > DNT. The difference of the three treatments all reached significant level (*P* < 0.05). DT > CT indicated that deep tillage increased the abundance of soil microorganism. It may be because deep tillage loosens the soil and adds the organic matter into the soil. Studies also proved that deep tillage could improve the soil physical characteristics, decrease the soil penetration resistance, and increase the soil porosity [17]. DT > DNT indicated that straw returning increased the abundance of soil microorganism. What is more, straw returning was proved to increase the carbon resource accelerating soil microorganism breeding and also straw increased the capacity of the small-sized fractions to protect soil microorganisms [18]. Govaerts et al. reported that residue retention induced higher population counts of total bacteria, fluorescent* Pseudomonas*, and actinomycetes compared to residue removal under zero tillage and conventional tillage [19].

#### 3.1.1. Differences of Two Soil Textures

Soil microbes were affected by soil texture. The number of soil actinomycetes, bacteria, and fungi in loam and clay soil was shown in Figures 1, 2, and 3 and Table 1. The significant differences between the loam and clay soil were found (*P* < 0.05). It was shown that the number of soil actinomycetes and fungi in clay soil was 151.2% and 42.9% higher than those in loam soil. Maybe it was because clay soil contained more fine texture clay particles than loam soil. Meliani et al. revealed that fine-textured soils typically contain greater quantities of organic matter and microbial biomass than coarse-textured soils [20]. Clay-sized particles were thought to shelter soil microorganisms from predation [21]. According to the study of Alvarez et al., the fine-textured soil (<50 microns) has a protective effect on total microbial biomass [22].

#### 3.1.2. Differences of Three Microbe Species

For actinomycetes, bacteria, and fungi, the DTCT values were calculated from (1). DT treatment in loam was higher than CT by 52.3%, 16.4%, and 14.6%, while in clay, DT treatment was higher than those of CT by 24.3%, 14.5%, and 31.3%. These indicated that deep tillage had the greatest effect on soil actinomycete in loam and on the soil fungi in clay. For soil bacteria, effect of deep tillage in clay was a bit higher than in loam:

(1) $$\begin{array}{c} \text{DTCT}=\frac{\text{DT}-\text{CT}}{\text{CT}}. \end{array}$$

Compared with DNT, from the DTDNT value (2), the number of actinomycetes, bacteria, and fungi of DT treatment was greatly increased by 77.0%, 50.0%, and 158.2% in loam, while those of DNT treatment in clay were increased by 177.5%, 62.7%, and 54.0%. These suggested that straw returning increased the number of soil fungi, bacteria, and actinomycetes. The effect of straw returning on actinomycete and bacteria in clay was greater than that in loam. Effect of straw returning on soil fungi abundance in clay was lower than that in loam soil:

(2) $$\begin{array}{c} \text{DTDNT}=\frac{\text{DT}-\text{DNT}}{\text{DNT}}. \end{array}$$

#### 3.1.3. Differences of Soil Depth

Figures 1, 2, and 3 showed that no matter clay or loam soil, with the soil sampling depth increasing, the number of soil actinomycetes, bacteria, and fungi decreased constantly. This agreed with the result of Qiao et al. [23]. And Zhao also found that bacteria, fungi, and actinomycetes mainly distribute in the layer of 0–40 cm, being gradually decreased with the increasing depth of soil layers [24]. The surface soil contained more soil microorganisms than the deep soil. Maybe there were more organic matter or organic carbon and dissolved organic carbon providing good living condition and food for soil microorganism in the surface soil [25].

Tillage management greatly affected the distribution of soil microorganism in different soil depths. Seen from Table 2, DT treatment had the greatest effect on actinomycete in the depth of 20–30 cm, which can be obtained from the value of DTDNT in different depths. Similarly, in the depth of 20–30 cm, DT treatment contained higher bacteria and fungi numbers than CT. These data all suggested that soil microorganism at the soil depth of 20–30 cm was more easily affected by deep tillage. Egamberdiyeva et al. also reported that microbial population was different in different soil depths. The abundance of bacteria was found higher at 20–30 cm depth after tomato and wheat tillage [26].

Straw returning was another factor affecting the soil microorganism in different soil depths. Seen from Table 2, actinomycete numbers in the depth of 20–30 cm were easier to be influenced by straw returning. This could be indicated by the highest DTDNT values, which were 111.1% and 308.3% higher in loam and clay, respectively. For bacteria, the depths of 10–20 cm in loam and 20–30 cm in clay were the easiest affected layer, with the highest DTDNT of 78.5% and 99.8%. And for fungi, the depths of 0–10 cm in loam and 10–20 cm in clay were the easiest affected layer, with the highest DTDNT of 234.2% and 104.5%. These results showed that soil microorganisms in the depth of 0–30 were the most easily affected layer by straw returning.

### 3.2. Effects of Deep Tillage and Straw Returning on Soil Enzyme Activities

**Figure 4:**
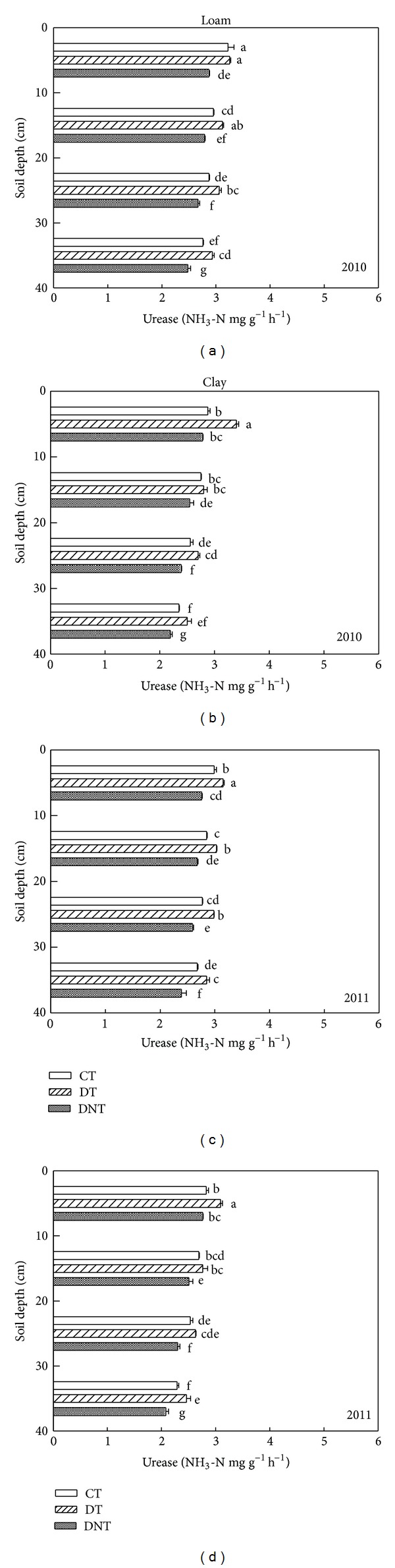
Effects of tillage and straw returning on soil urease activity. Different small letters indicated significant difference at *P* < 0.05 according to Duncan's test. CT represented conventional tillage, moldboard ploughed to a depth of 20 cm, and straw was returned. DT represented deep tillage moldboard ploughed to a depth of 30 cm and straw was returned. DNT represented deep tillage moldboard ploughed to a depth of 30 cm and straw was removed.

**Figure 5:**
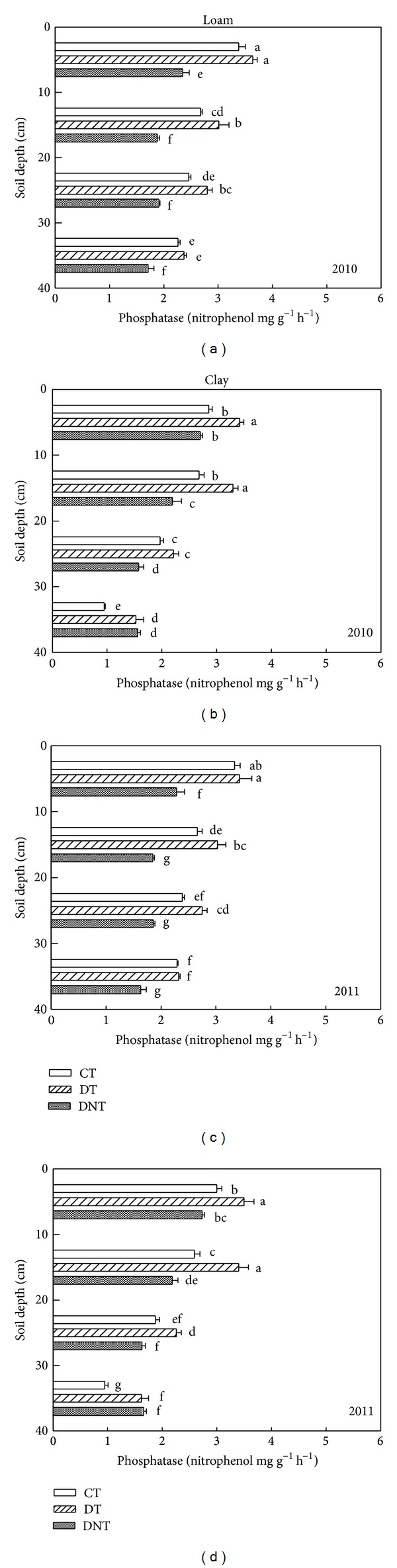
Effects of tillage and straw returning on soil phosphatase activity. Different small letters indicated significant difference at *P* < 0.05 according to Duncan's test. CT represented conventional tillage, moldboard ploughed to a depth of 20 cm, and straw was returned. DT represented deep tillage moldboard ploughed to a depth of 30 cm and straw was returned. DNT represented deep tillage moldboard ploughed to a depth of 30 cm and straw was removed.

**Figure 6:**
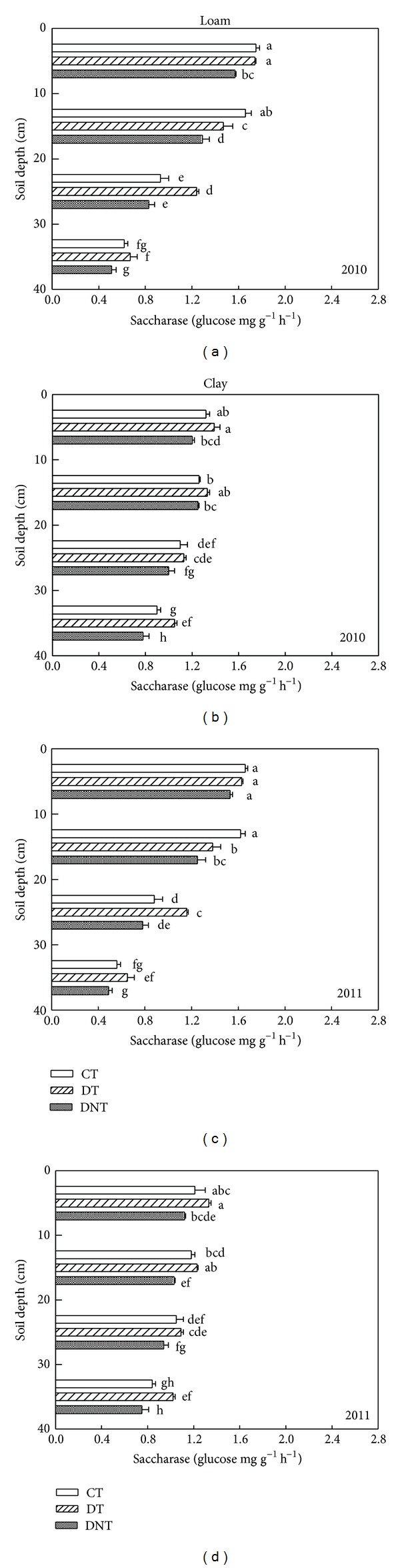
Effects of tillage and straw returning on soil saccharase activity. Different small letters indicated significant difference at *P* < 0.05 according to Duncan's test. CT represented conventional tillage, moldboard ploughed to a depth of 20 cm, and straw was returned. DT represented deep tillage moldboard ploughed to a depth of 30 cm and straw was returned. DNT represented deep tillage moldboard ploughed to a depth of 30 cm and straw was removed.

**Figure 7:**
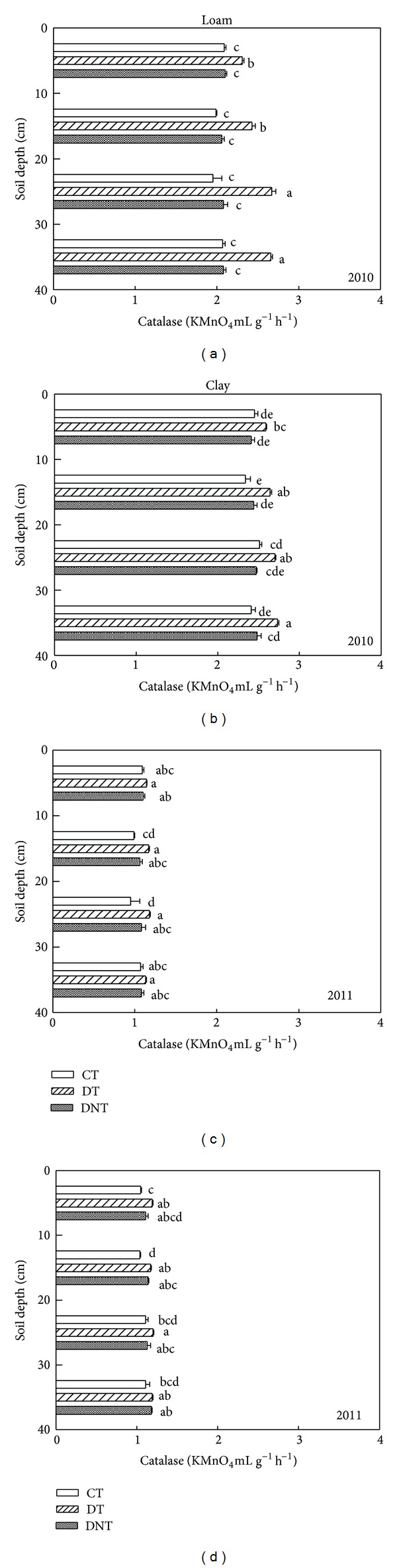
Effects of tillage and straw returning on soil catalase activity. Different small letters indicated significant difference at *P* < 0.05 according to Duncan's test. CT represented conventional tillage, moldboard ploughed to a depth of 20 cm, and straw was returned. DT represented deep tillage moldboard ploughed to a depth of 30 cm and straw was returned. DNT represented deep tillage moldboard ploughed to a depth of 30 cm and straw was removed.

Tillage and straw returning had great effects on soil enzyme activities. The soil enzymes activities (urease, phosphatase, saccharase, and catalase) of CT, DT, and DNT treatments in loam and clay were shown in Figures 4, 5, 6, and 7. Seen from the figures and Table 1, the activities of urease, phosphatase, and saccharase generally followed the order of DT > CT > DNT. For the four soil enzyme activities, it had the tendency of DT > CT, which indicated that deep tillage increased the soil enzyme activities. It may be because deep tillage loosens the soil and adds the organic matter into the soil, which increased the abundance of soil microorganism. The more soil microorganism, the higher soil enzyme activities. It was consistent with our results of deep tillage on soil microorganism. On the other hand, urease, phosphatase, and saccharase activities of DT were higher than DNT. This suggested that straw returning increased most soil enzyme activities. Jin et al. also reported that subsoiling with mulch consistently had higher enzyme activities compared with no-till with mulch [27].

#### 3.2.1. Differences of Two Soil Textures

Soil texture had great effect on soil enzyme activities. The activities of soil urease, phosphatase, saccharase, and catalase in loam and clay soil were shown in Figures 4, 5, 6, and 7 and Table 1. It could be found that the activities of urease in loam soil were 9.37% higher than those in clay soil. Phosphatase and catalase activities of clay soil were 9.85% and 9.60% higher than those of loam soil. Urease and catalase activities were significantly different between loam and clay soil. There was no obvious difference of saccharase activities between loam and clay soil. It suggested that most soil enzyme activities were higher in clay soil. Maybe it was because clay soil contains more fine texture clay particles inhabiting more soil microorganisms, which were the resources of soil enzymes [21, 22].

Seen from Table 3, DTNT values of urease, phosphatase, and saccharase activities in loam were lower than those in clay. It indicated that effect of deep tillage on most soil enzyme activities in the loam was lower than that in the clay soil. Similarly, DTDNT values of studied enzyme activities in the loam were higher than in clay. It suggested that effect of straw returning on the soil enzyme activities in the loam was higher than in the clay.

#### 3.2.2. Differences of Four Enzyme Species

Seen from the average values of each enzyme activity in clay and loam soil, the four enzyme activities followed the order of urease > phosphatase > catalase > saccharase. Of the average of all soil depths, urease activity of clay was lower than that of loam by 8.6% and the difference reached significant level (*P* < 0.05). Activities of phosphatase and catalase in clay soil were higher than those of loam soil by 10.9% and 10.6, respectively. The difference of catalase activity between clay and loam reached significant level (*P* < 0.01). Saccharase activity in clay was lower than in loam by 4.6%. Almost no difference was found between the loam and clay for the saccharase activity. It indicated that phosphatase and catalase enzyme activities were higher, and urease had lower activities in clay. Small and no significant difference of saccharase activities was found between the loam and clay soil. Activities of enzyme species may be related to organic matter mineralization and humification in the soil.

For the four soil enzyme activities regardless of soil texture, they had the similar tendency of DT > CT. Urease, phosphatase, and saccharase activities of DT were higher than DNT. But catalase activity of DT in the loam and clay was lower than DNT. They suggested that straw returning increased the activities of urease, phosphatase, and saccharase but decreased the catalase activities. The effect of straw returning on urease was in agreement with Lu et al. [28], which showed that the treatments incorporated with straw were higher in urease and phosphatase activities.

#### 3.2.3. Differences of Soil Depth

Effect of tillage on soil enzyme activities was affected by soil depth. With the increasing of soil depth, urease, saccharase, and phosphatase activities all decreased (Figures 4, 5, and 6). Kheyrodin et al. [29] also reported that urease activity decreased markedly with soil depth. Deng and Tabatabai [12] found that phosphatases activities decreased with increasing soil depth. They thought this decrease may be associated with the decrease in organic carbon content. Most enzyme activities in the surface soil were higher than deep soil. This may be because there were more soil microorganism and plant residues in the surface soil, which were the main parts of soil enzymes.

For urease, deep tillage in loam was most effective in the depth of 20–30 cm (Figure 4 and Table 3). At this soil depth, urease of DT had the highest DTCT value and was 6.95% higher than CT. For urease in clay soil, deep tillage was most effective in 0–10 cm. At this depth, urease activities of DT were 13.5% higher than CT. Similarly, deep tillage had the greatest effect on the activities of phosphatase, saccharase, and catalase at the depth of 20–30 cm in loam. For the clay, at the depth of 20–30 cm, deep tillage had the greatest effect on phosphatase and catalase activities. For saccharase in clay, it was at the depth of 30–40 cm where DT was 35.04% higher than CT. It suggested that deep tillage mainly affected the loam soil at the soil depth of 20–30 cm and the clay soil at almost all the studied soil depths from 0 to 40 cm.

Seen from Table 3, in loam, the greatly affected depth of the activities of urease, phosphatase, saccharase, and catalase by straw returning was 30–40 cm, 10–20 cm, 20–30 cm, and 20–30 cm. In clay, the greatest affected depth for urease, phosphatase, saccharase, and catalase activities was 0–10 cm, 20–30 cm, 30–40 cm, and 20–30 cm. It suggested that straw returning affected soil enzyme activities at almost all the soil depths (0–40 cm).

## 4. Conclusions

In conclusion, deep tillage and straw returning increased the abundance of soil microorganism and most enzyme activities. Deep tillage was more effective for increasing enzyme activities in clay, while straw returning was more effective in loam. Soil microorganism abundance and most enzyme activities decreased with the increase of soil depth. Deep tillage mainly affected soil enzyme activities in loam at the soil depth of 20–30 cm and in clay soil at the depth of 0–40 cm. Straw returning mainly affected soil microorganism and soil enzyme activities at the depths of 0–30 cm and 0–40 cm, respectively.

## Figures and Tables

**Table 1:** Differences of tillage, year, soil depth, and soil texture on soil microorganism and enzyme activities.

Soil texture	Factor		Actinomycete	Bacteria	Fungi	Urease	Phosphatase	Saccharase	Catalase
Loam	Y (year)	2010	1.02	4.64^a^	1.55^a^	2.91^a^	2.54	1.19^a^	2.21^a^
		2011	0.92	1.56^b^	1.06^b^	2.81^b^	2.49	1.13^b^	1.09^b^
	T (tillage)	CT	0.86^b^	3.17^b^	1.51^b^	2.89^b^	2.11^b^	1.21^a^	1.53^c^
		DT	1.31^a^	3.69^a^	1.73^a^	3.05^a^	2.65^a^	1.24^a^	1.58^b^
		DNT	0.74^c^	2.46^c^	0.67^c^	2.65^c^	2.03^b^	1.03^b^	1.83^a^
	*D* (soil depth)	0–10 cm	1.71^a^	4.62^a^	2.92^a^	3.04^a^	3.04^a^	1.65^a^	1.64^a^
		10–20 cm	1.17^b^	3.58^b^	1.43^b^	2.90^b^	2.72^b^	1.45^b^	1.62^ab^
		20–30 cm	0.75^c^	2.97^c^	0.57^c^	2.82^c^	1.92^c^	0.97^c^	1.65^ab^
		30–40 cm	0.26^d^	1.24^d^	0.28^d^	2.68^d^	1.38^d^	0.58^d^	1.68^b^

Clay	Y (year)	2010	2.33^b^	2.79^b^	1.88	2.65^a^	2.25	1.16^a^	2.51^a^
		2011	2.83^a^	3.36^a^	1.83	2.58^b^	2.28	1.07^b^	1.13^b^
	T (tillage)	CT	2.88^b^	3.24^b^	1.76^b^	2.68^a^	2.68^b^	1.11^b^	1.75^c^
		DT	3.58^a^	3.71^a^	2.31^a^	2.72^a^	2.92^a^	1.19^a^	1.79^b^
		DNT	1.29^c^	2.28^c^	1.50^c^	2.44^b^	1.93^c^	1.04^c^	1.93^a^
	*D* (soil depth)	0–10 cm	4.19^a^	5.46^a^	3.73^a^	2.96^a^	3.07^a^	1.26^a^	1.80^b^
		10–20 cm	3.76^b^	3.76^b^	2.09^b^	2.67^b^	2.52^b^	1.24^a^	1.79^b^
		20–30 cm	1.73^c^	2.27^c^	1.16^c^	2.52^c^	2.36^c^	1.05^b^	1.85^a^
		30–40 cm	0.64^d^	0.82^d^	0.45^d^	2.31^d^	2.10^d^	0.89^c^	1.85^a^

Note: different small letters in the column indicated significant difference at *P* < 0.05. Differences between 2010 and 2011 were compared by the Student *t*-test (2-tailed test). Differences among the different soil depths and soil tillage were determined by Duncan's test. CT represented conventional tillage, moldboard ploughed to a depth of 20 cm, and straw was returned. DT represented deep tillage moldboard ploughed to a depth of 30 cm and straw was returned. DNT represented deep tillage moldboard ploughed to a depth of 30 cm and straw was removed.

**Table 2:** DTCT and DTDNT values of soil microorganism in the loam and clay soil.

	Depth	Actinomycete		Bacteria		Fungi	
		Loam	Clay	Loam	Clay	Loam	Clay
DTCT	0–10 cm	0.447	0.251	0.041	0.003	0.057	0.099
	10–20 cm	0.628	0.241	−0.024	0.062	0.197	0.201
	20–30 cm	**0.797 **	**0.301 **	**0.707 **	**0.671 **	**0.613 **	**1.987 **
	30–40 cm	0.030	0.064	0.428	0.423	0.203	0.401

DTDNT	0–10 cm	0.648	1.660	0.749	0.366	**2.342 **	0.248
	10–20 cm	1.032	1.663	**0.785 **	0.839	1.702	**1.045 **
	20–30 cm	**1.111 **	**3.038 **	0.198	**0.998 **	0.486	0.841
	30–40 cm	0.008	0.973	0.106	0.686	0.058	0.587

Bold numbers in the column represented the maximal value.

**Table 3:** DTCT and DTDNT values of urease, phosphatase, saccharase, and catalase activities in loam and clay soil.

		Urease		Phosphatase		Saccharase		Catalase	
		Loam	Clay	Loam	Clay	Loam	Clay	Loam	Clay
DTCT	0–10 cm	0.031	**0.135 **	0.051	0.182	−0.012	0.077	0.076	0.091
	10–20 cm	0.058	0.021	0.132	0.273	−0.131	0.047	0.197	**0.127 **
	20–30 cm	**0.070 **	0.045	**0.144 **	0.159	**0.321 **	0.030	**0.305 **	0.079
	30–40 cm	0.062	0.070	0.031	**0.659 **	0.123	**0.183 **	0.171	0.107

DTDNT	0–10 cm	0.137	**0.169 **	0.524	0.274	0.087	0.175	0.066	0.070
	10–20 cm	0.124	0.099	**0.622 **	0.533	0.119	0.122	0.145	0.056
	20–30 cm	0.148	0.136	0.472	**0.388 **	**0.488 **	0.144	**0.188 **	**0.076 **
	30–40 cm	**0.188 **	0.164	0.405	−0.025	0.322	**0.350 **	0.160	0.058

Bold numbers in the column represented the maximal value.
